# Platelet‐Rich Fibrin Induces New Bone Formation to Promote Extreme Lateral Interbody Fusion of Spine in Rabbits

**DOI:** 10.1002/jsp2.70179

**Published:** 2026-04-15

**Authors:** Weijian Wang, Jiaqi Li, Han Wu, Sidong Yang, Shaorong Li, Wei Zhang

**Affiliations:** ^1^ The Third Department of Spine The Third Hospital of Hebei Medical University Shijiazhuang China

**Keywords:** allogeneic bone, artifificial bone, extreme lateral interbody fusion, fusion outcome, platelet‐rich fibrin, rabbit

## Abstract

**Background:**

Achieving rapid bone fusion is critical for preventing complications in Extreme Lateral Interbody Fusion (XLIF), yet harvesting sufficient autologous bone presents surgical limitations. Platelet‐rich fibrin (PRF), an autologous biomaterial rich in osteogenic growth factors, offers potential as a bone graft enhancer. This study evaluated the efficacy of PRF combined with allogeneic bone in promoting interbody fusion using an XLIF‐simulated rabbit model.

**Methods:**

In vitro, bone marrow mesenchymal stem cells were cultured with PRF, allogeneic bone, or PRF/allogeneic bone composites. Assessments included biocompatibility (CCK‐8, Calcein‐AM/PI), cell adhesion (phalloidin/DAPI), and osteogenic differentiation (alkaline phosphatase activity/staining, Alizarin Red S). PRF, allogeneic bone, and their composite (PRF/allogeneic bone) were evaluated in a rabbit XLIF model. Autologous iliac crest bone served as a positive control, while empty cages provided negative controls. Endpoints included radiographic (micro‐CT), mechanical (biomechanical testing), histological (H&E, methylene blue–acid fuchsin, TRAP), and biochemical (ELISA) evaluation at postoperative 2, 4, 8, and 12 weeks.

**Results:**

In vitro experiments demonstrated that PRF/allogeneic bone composites exhibited noncytotoxic properties and osteogenic‐promoting effects when combined with titanium alloy cages. In vivo, fusion progressed temporally across all groups, with the PRF/allogeneic bone composite yielding 12‐week fusion rates by manual palpation and micro‐CT equivalent to autograft. Biomechanical strength and bone mineral density of PRF/allogeneic bone matched autograft, exceeding allogeneic bone. Histology demonstrated accelerated fusion kinetics: early angiogenesis (2 weeks), fibrocartilage formation (4 weeks), and complete trabecular bridging by 12 weeks. ELISA confirmed earlier BMP‐2/VEGF peaks (2–4 weeks) versus allogeneic bone.

**Conclusions:**

These results indicated that the combination of PRF and allogeneic bone successfully induced intervertebral bone formation in the rabbit XLIF model. PRF can serve as a physiological natural fusion material, inducing osteogenesis and achieving spinal fusion. Its osteopromotive effects, cost‐effectiveness, and autologous origin support its potential as a superior graft alternative for XLIF procedures.

## Graphical abstract

Preparation process of PRF and its promotion of intervertebral fusion. The rabbit lateral fusion model proves that PRF can promote the intervertebral fusion rate of allograft bone. In vitro experiments can be observed that PRF promotes the osteogenic expression of BMSCS.

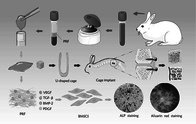

## 1 Introduction

Extreme Lateral Lumbar Interbody Fusion (XLIF) is a minimally invasive surgical approach performed through the lateral retroperitoneal space. Its core advantage lies in blunt dissection of the psoas major muscle to access the lateral intervertebral space, followed by implantation of a large interbody cage to achieve indirect decompression and interbody fusion. Compared to traditional posterior lumbar surgery, XLIF significantly reduces intraoperative muscle damage, shortens operative time, minimizes blood loss, and preserves the stability of the lumbar posterior column and vertebral structure, establishing it as a revolutionary technique widely adopted in spinal surgery [1, 2].

Although autologous bone remains the “gold standard” for grafting due to its superior osteoinductive properties, the inability to harvest sufficient autologous bone within the XLIF surgical field necessitates additional bone extraction, increasing surgical morbidity and complication risks. Critically, XLIF relies on a large cage to distract the intervertebral space for indirect decompression. Delayed or failed postoperative bone fusion may lead to severe complications such as intervertebral instability, cage displacement, intervertebral space collapse, and implant failure, directly compromising surgical outcomes. Bone fusion efficacy is pivotal to the long‐term success of XLIF. Therefore, developing high‐performance fusion materials to replace or surpass autologous bone has become a central research focus for optimizing clinical results in XLIF.

Platelet‐Rich Fibrin (PRF), a second‐generation platelet concentrate (first reported by Choukroun et al. [3]), offers advantages over traditional platelet‐rich plasma (PRP), including simplified preparation and no requirement for anticoagulants. Its gel matrix is rich in autologous growth factors that synergistically promote bone regeneration and soft tissue repair [4, 5, 6]. With its autologous origin, low cost, and easy accessibility, PRF emerges as an ideal bone fusion‐enhancing material for XLIF. Through in vitro experiments and a New Zealand rabbit lateral interbody fusion model, this study systematically evaluates the comprehensive effects of PRF combined with bone grafts on pathological tissue remodeling, radiographic fusion, and proteomic profiles, aiming to provide a theoretical foundation for optimizing bone graft selection in XLIF procedures.

## 2 Materials and Methods

### 2.1 Materials

#### 2.1.1 Manufacturing of Titanium Alloy Cages and Titanium Disks

The titanium alloy (Ti6Al4V) U‐shaped cages (10 mm length × 2.5 mm width × 1.3 mm height) were custom‐designed and fabricated using medical‐grade titanium powder (AK Medical Holdings Limited, Beijing, China) [7]. Titanium disks are trimmed from U‐shaped cage heads, suitable in size for cell culture plates.

Autologous bone graft: Iliac crest bone graft was harvested from the same rabbit undergoing surgery. The Cancellous autograft bone was morselized with manual instruments into small pieces (< 5 mm).

Allogeneic bone graft: Iliac crest bone was harvested from additional New Zealand White rabbits euthanized for unrelated, approved studies. Bone particles were prepared to match the autologous bone graft size specifications (< 5 mm). The particles were processed by sequential washing in sterile saline, defatting in a 1:1 chloroform‐methanol solution for 4 h, deproteinization in 30% hydrogen peroxide for 24 h, and surface decalcification in 0.6 N HCl for 10 min. Processed particles were lyophilized, vacuum‐sealed, and sterilized by gamma irradiation (^60^Co, 20 kGy).

PRF preparation: 4 mL of whole blood was drawn from the marginal ear vein using a needle directly into a sterile, plain glass‐coated plastic vacuum tube (without anticoagulant). The tube was immediately centrifuged (Rotor Radius 10 cm) at 3000 rpm for 10 min, yielding a relative centrifugal force of approximately 400 × g [8]. After centrifugation, three layers formed: acellular plasma (top), PRF clot (middle), and red blood cells (bottom). The PRF clot was separated from the lower red blood cell layer using sterile scissors and gently compressed between sterile gauze to form a membrane. For in vitro experiments, PRF was prepared from a separate donor rabbit and processed identically. The resulting PRF membrane was cut into small fragments (1 × 1 mm) using a scalpel.

PRF/Allogeneic Bone Composite: The prepared allogeneic bone particles were mixed with the freshly prepared PRF fragments at a 1:1 volume ratio immediately prior to implantation. The mixture was gently combined to ensure uniform distribution.

### 2.2 In Vitro Research Cell Experiments

#### 2.2.1 Isolation and Culture of Bone Marrow Mesenchymal Stem Cells

Bone marrow mesenchymal stem cells (BMSCs) were obtained from long bone marrow from New Zealand rabbits. BMSCs were cultured in low‐glucose DMEM containing 1% streptomycin–penicillin and 10% FBS in a humidified incubator at 37°C and 5% CO2. Cells were digested and passaged at approximately 80% confluence. The third passage cells were used for in vitro experiments.

#### 2.2.2 Experimental Groups and Cell Culture

Before co‐culturing with cells, four experimental groups were established on titanium disks placed in cell culture plates: (1) the Allograft group (loaded with 100 mg allogeneic bone particles), (2) the PRF group (loaded with 100 mg PRF fragments sized 10–20 mm [2]), (3) the PRF/Allograft group (loaded with a mixture of 100 mg allogeneic bone particles and an equivalent amount of PRF fragments), and (4) the Blank group (disks with no material loading).

#### 2.2.3 Biocompatibility

CCK‐8 assays were performed to study cell proliferation. The proliferation of BMSCs co‐cultured with the various materials was at a density of 1 × 10 [4] cells/well in a 24‐well culture plate. Subsequently, the proliferation of cells adherent to the materials was measured for 1, 3, and 7 days using the CCK‐8 assay according to the manufacturer's instructions (Beyotime Biotechnology, Shanghai, China). Calcein‐AM/PI staining was performed after 1 and 3 days of culture to assess cell viability in each group, according to the manufacturer's protocol. The samples were then imaged using a confocal laser scanning microscope (LSM780, ZEISS, Germany). Green‐stained cells indicate live cells, while red‐stained cells indicate dead cells.

#### 2.2.4 Cell Attachment

Cells were fixed with 3.7% paraformaldehyde, stained with Actin‐Tracker Red‐555 (Beyotime Biotechnology, Shanghai, China) and DAPI (Beyotime Biotechnology, Shanghai, China), and observed under a confocal laser scanning microscope (LSM780, ZEISS, Germany).

#### 2.2.5 Alizarin Red S (ARS) Staining

BMSCs were cultured onto the materials from each experimental group and BMP‐2 group (100 ng/mL) in a 24‐well plate at a density of 1 × 10 [5] cells/well. After cells adhered, the medium was replaced with osteogenic induction medium. The medium is changed every 3 days. Osteogenic differentiation ability was assessed at 14 and 21 days. At predetermined time points, cells were fixed with 4% paraformaldehyde at 37°C for 30 min and washed 3 times with PBS. The ARS solution was added to the sample at room temperature for 20 min. After washing with PBS to remove residual stains, a microscope (Olympus IX.) Take an image. To quantify the results of ARS, samples were dissolved in 10% cetylpyridinium chloride and the color of each sample was read at 560 nm by a microplate reader.

#### 2.2.6 Alkaline Phosphatase (ALP) Staining and Activity Assay

Cells were prepared by washing with PBS to remove residual culture medium, followed by fixation with 4% paraformaldehyde for 10 min at room temperature. After fixation, samples were rinsed thoroughly with PBS. The staining solution prepared with BCIP/NBT (5‐bromo‐4‐chloro‐3‐indolyl‐phosphate/nitro blue tetrazolium) in alkaline buffer was applied to cover the samples. The reaction was performed in the dark at 37°C for 30 min. Staining progression was monitored visually, and the reaction was terminated by PBS washing. ALP‐positive regions were examined under a light microscope, and images were quantitatively analyzed using ImageJ software.

To determine alkaline phosphatase activity, the culture medium was removed at 7 and 14 days, and cells were washed with PBS. Subsequently, cells were permeabilized with 0.1% Triton X‐100 to induce lysis. The lysate was centrifuged to obtain supernatant. Alkaline phosphatase activity was assessed using an ALP Assay Kit (Beyotime, Shanghai, China), while total protein per well was measured with a BCA Protein Assay Kit (Beyotime, Shanghai, China). ALP activity was normalized to total intracellular protein content.

### 2.3 In Vivo Osseointegration

#### 2.3.1 Experimental Animals and Ethical Approval

This study used 100 skeletally mature female New Zealand White rabbits, weighing between 2.2–2.5 kg (average 2.4 kg), all raised under the same housing conditions. The rabbits were randomly divided into five groups: Group A (Autologous Iliac Bone, *n* = 10), Group B (Allogeneic Bone, *n* = 25), Group C (PRF/Allogeneic Bone, *n* = 40), Group D (PRF, *n* = 20), and Group E (Blank, Empty cages, *n* = 5). Sample sizes were based on well‐accepted group sizes for comparative biomaterials evaluation in this model as previously published and were weighted toward the primary fusion endpoint at 12 weeks [7, 9]. At 12 weeks postoperation, the surgical segments of groups A, B, D, C, and E were subjected to manual palpation, Micro‐CT, biomechanical testing to evaluate fusion status. HE, methylene blue‐acid fuchsin staining, and Trap staining were used to observe the pathological changes under different fusion materials at 12 weeks postoperation in groups A and B, and at 2, 4, 8, and 12 weeks postoperation in group C. In groups B, C, and D, ELISA was performed to detect BMP‐2, TGFβ‐1, VEGF, and PDEG 2, 4, 8, and 12 weeks postoperation. Experimental groups are presented in Table S1.

In accordance with the National Institute of Health Guide, this study was approved by the Animal Ethics Committee of the Third Hospital of Hebei Medical University, under the protocol number Z 2022–001‐1. At predetermined time points, designated subjects from each group were humanely euthanized via intracardiac injection of pentobarbital sodium under deep anesthesia, followed by surgical excision of spinal segments.

#### 2.3.2 Extreme Lateral Interbody Fusion Surgical Procedure

**FIGURE 1:**
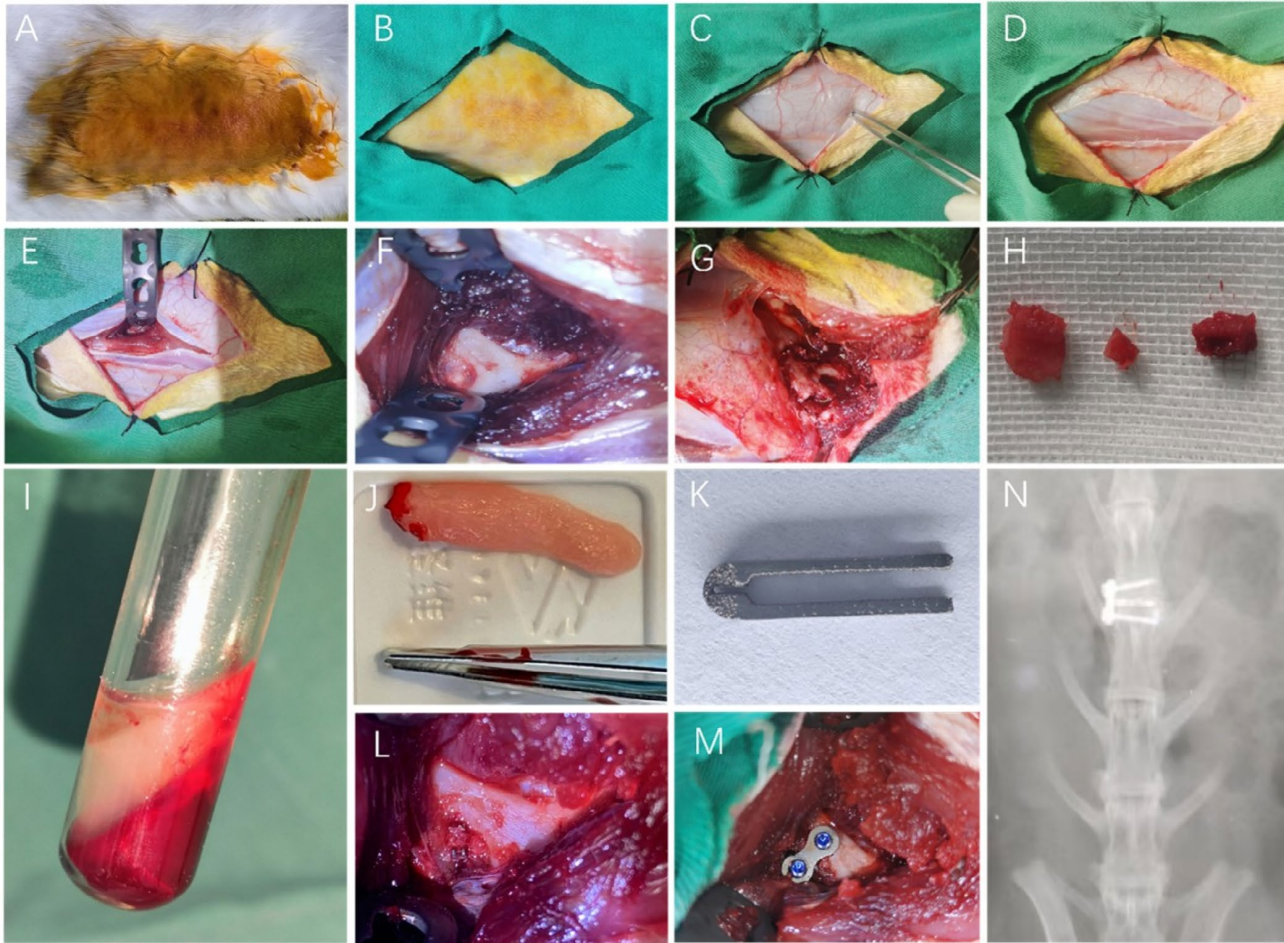
Surgical procedure. (A) Rabbit lumbar vertebrae lateral shaving and disinfection. (B) Surgical area draping. (C) Cutting the skin. (D) Cutting the fascia. (E) Blunt dissection of paraspinal muscles after opening the muscle fascia. (F) Stripping of lateral lumbar bone muscle. (G) Taking the iliac bone block on the same side. (H) The appropriate amount of autologous iliac bone removed was used for bone grafting. (I) Blood was drawn from the rabbit ear vein and centrifuged. (J) PRF can be seen in the middle layer of the centrifuge tube (K) Self—designed titanium alloy U‐shaped cage. (L) The cage was placed in the intervertebral space and then filled with different bone graft materials. (M) The surgical segment was fixed with lumbar lateral screw plate. (N) Postoperative anteroposterior x‐ray.

After isoflurane inhalation anesthesia, the rabbits were placed in the right lateral position. Following shaving and disinfection, an incision was made with the L4‐5 intervertebral space as the center. The lateral sides of the L4 and L5 vertebrae were exposed by dissecting along the outer edges of the sacrospinal and quadratus lumborum muscles and the fascial space. After disrupting the annulus fibrosus, the nucleus pulposus was removed. The titanium alloy U‐shaped cage was first implanted into the intervertebral space, followed by the placement of the corresponding bone graft materials within the cage, with the implantation quantity determined by filling the cage to capacity. A two‐hole fixation plate was placed laterally on the surgical segment, and a screw was inserted into the vertebral bodies on both the cranial and caudal sides. After thorough irrigation, the wound was sutured layer by layer and covered with sterile dressings. Penicillin (40 000 U/kg) was administered intramuscularly for 3 days postsurgery to prevent infection (Figure 1).

#### 2.3.3 Manual Palpation Analysis

Ten rabbits from the groups A, B, C and 5 rabbits from the groups D and E were euthanized 12 weeks postsurgery. After sampling, the muscles around the lumbar spine were removed, retaining the supraspinous ligament, interspinous ligament, and articular processes. The fibrous tissue around the bone graft was carefully removed with a sharp knife, and the internal fixation nail plate was extracted. Three experienced and blinded spinal surgeons who did not participate in the surgeries manually checked the hardness of the bone graft and assessed the flexion and extension range of motion of the fusion gap. If no range of motion was detected, the fusion was considered firm; otherwise, it was deemed unfused. The final call required agreement by at least two of the three surgeons.

#### 2.3.4 Micro‐Computed Tomography Analysis

Twelve weeks after surgery, the ribs of the lumbar specimens were trimmed, and Micro‐CT scans were performed using the SkyScan 1173 (Bruker, Belgium) with the following scanning parameters: rotation angle 360°, rotation angle increment 0.4°, scanning resolution 14 μm, frame averaging 4, pixel combination 1 × 1, exposure time 3000 ms, voltage 80 kV, and current 80 μA.

N‐Recon software was used to perform three‐dimensional bone reconstruction of the sample images. The internal area of the U‐shaped fusion cage in lumbar surgery was measured and analyzed using CT‐AN software. The selection of ROI was based on cage visualization, where the central portion of the U‐shaped cage was manually chosen to calculate total bone volume (BV). At 8 and 12 weeks postsurgery, the trabecular thickness (Tb.Th), trabecular number (Tb.N), bone volume fraction (BV/TV, BVF), and bone mineral density (BMD) of the specimens were measured.

#### 2.3.5 Biomechanical Testing

The specimens were trimmed of fat, muscle, and other soft tissues, retaining ligaments, joint capsules, intervertebral disks, and complete bony structures. The fused specimens were placed on two supports of a three‐point bending apparatus (TA. ElectroForce, Bose Corporation, USA), and a vertically downward force was applied to the intervertebral space of the surgical segment. The loading device descended at a constant speed of 0.5 mm/s until the vertebrae above and below the fused segment separated. A load–displacement curve was obtained, and the maximum pressure at the point of fusion failure was recorded, thereby determining the biomechanical parameters of the intervertebral fusion area.

#### 2.3.6 Histological Examination

Five samples from the lumbar surgical segment of Group A and Group B were collected 12 weeks postoperation, and five samples of Group C were collected at 2, 4, 8, and 12 weeks postoperation, with soft tissue removed. The fresh specimens were fixed in 4% paraformaldehyde for more than 24 h. Following fixation, the tissues were placed in EDTA decalcification solution until a needle could smoothly pass through the bone tissue. The lateral plate and fusion cage were then removed. The specimens were embedded in paraffin and sectioned to a thickness of 5 μm. Hematoxylin and eosin (H&E), methylene blue‐acid fuchsin, and Trap staining were performed to observe the pathological changes under different fusion materials using a light microscope.

#### 2.3.7 Elisa

Five samples were taken from each group at 2, 4, 8, and 12 weeks after operation in groups B, C, and D. Tissue samples from the fusion bed were harvested, snap‐frozen, and homogenized in RIPA buffer with protease inhibitors. Protein concentration was determined by BCA assay. All samples were normalized to total protein concentration (μg/ml) prior to analysis. ELISA kits for rabbit BMP‐2, VEGF, PDGF, and TGF‐β1 (Jingmei Biotechnology, Jiangsu, China) were used according to the manufacturer's protocols. Each sample (*n* = 3 biological replicates per group per time point) was assayed in technical duplicate.

### 2.4 Statistical Analysis

Statistical analysis was performed using SPSS 26.0 statistical software (IBM, Armonk, New York, USA). Measurement data were expressed as mean ± standard deviation (x̄±s). Data that were normally distributed and that had homogeneous variances was compared among groups using analysis of variance (ANOVA). For nonnormal data, the Kruskal‐Wallis test was used. All pairwise comparisons were adjusted using the Sidak method. Categorical data were described as percentages, and comparisons among groups were performed using the Chi‐square test (χ [2] test) or Fisher's exact test. A *p* value of < 0.05 was considered to indicate a statistically significant difference.

## 3 Results

### 3.1 In Vitro Research Cell Experiments

#### 3.1.1 Biocompatibility

**FIGURE 2:**
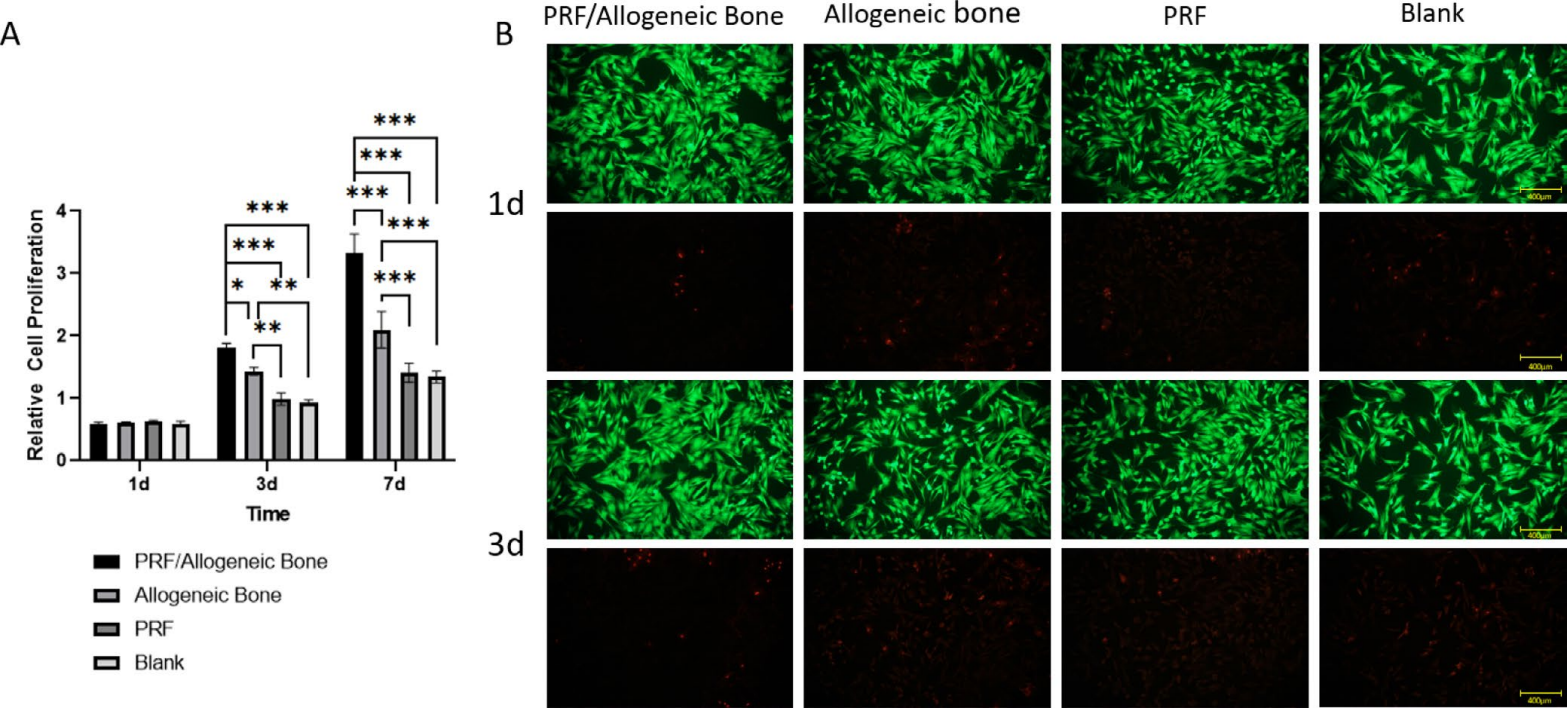
Biocompatibility. (A) CCK‐8 assays measure the cell proliferation of BMSCs in different groups. (B) Calcein‐AM/PI staining of BMSCs at day 1 and 3 (green represents live cells, whereas red represents dead cells). * indicates significant difference between groups, *p < 0.05, **p < 0.01, and ***p < 0.001.

We first evaluated the effects of different materials on cell proliferation using the CCK‐8 assay. As shown in Figure 2A, cell proliferation gradually increased across all groups over the 7‐day period. On day 1, there was no significant difference in the number of adherent cells among the groups. On posttreatment days 3 and 7, the PRF/allograft group exhibited significantly higher proliferation than the other groups (*p* < 0.05). Furthermore, Calcein‐AM/PI staining images revealed a large number of live cells (green fluorescence) and only a few dead cells (red fluorescence) in all groups on both day 1 and day 3, indicating good cell viability throughout (Figure 2B). The incorporation of PRF and allogeneic bone has no significant effect on the number of living cells, which proves that PRF and allogeneic bone are nontoxic and excellent biocompatible.

#### 3.1.2 Cell Attachment

**FIGURE 3:**
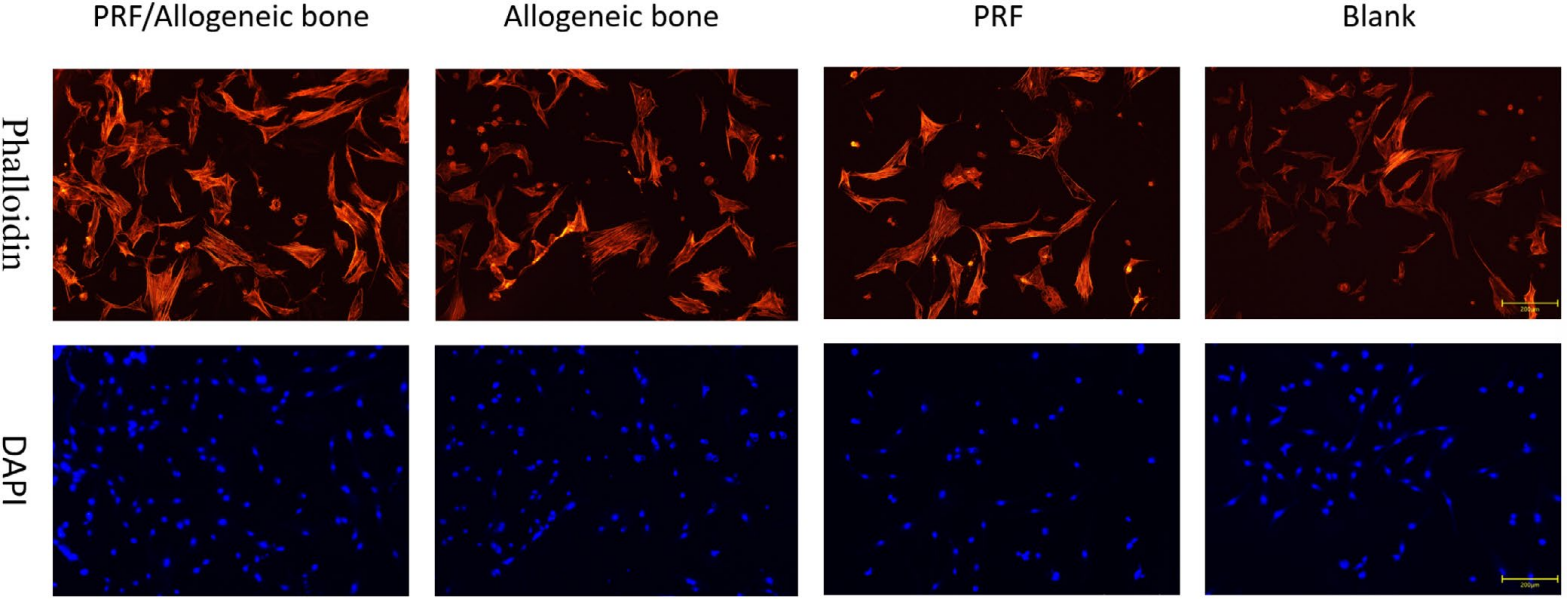
Staining of cytoskeleton. Phalloidin/DAPI staining. There were no significant differences in cytoskeleton staining among the four groups.

The morphology of BMSCs co‐cultured with the different material groups for 3 days was assessed using phalloidin/DAPI staining. Images of actin (red) and DAPI (blue) are shown in Figure 3. BMSCs on all groups displayed a uniform distribution and elongated morphology similar to fibroblasts. BMSCs exhibited more extensive spreading on the PRF/allograft group compared to the other groups. The increased number of BMSCs in the PRF/allograft group suggests that the surface may favor cell proliferation and adhesion compared to the other groups, demonstrating superior biocompatibility.

#### 3.1.3 Osteoblast Differentiation

**FIGURE 4:**
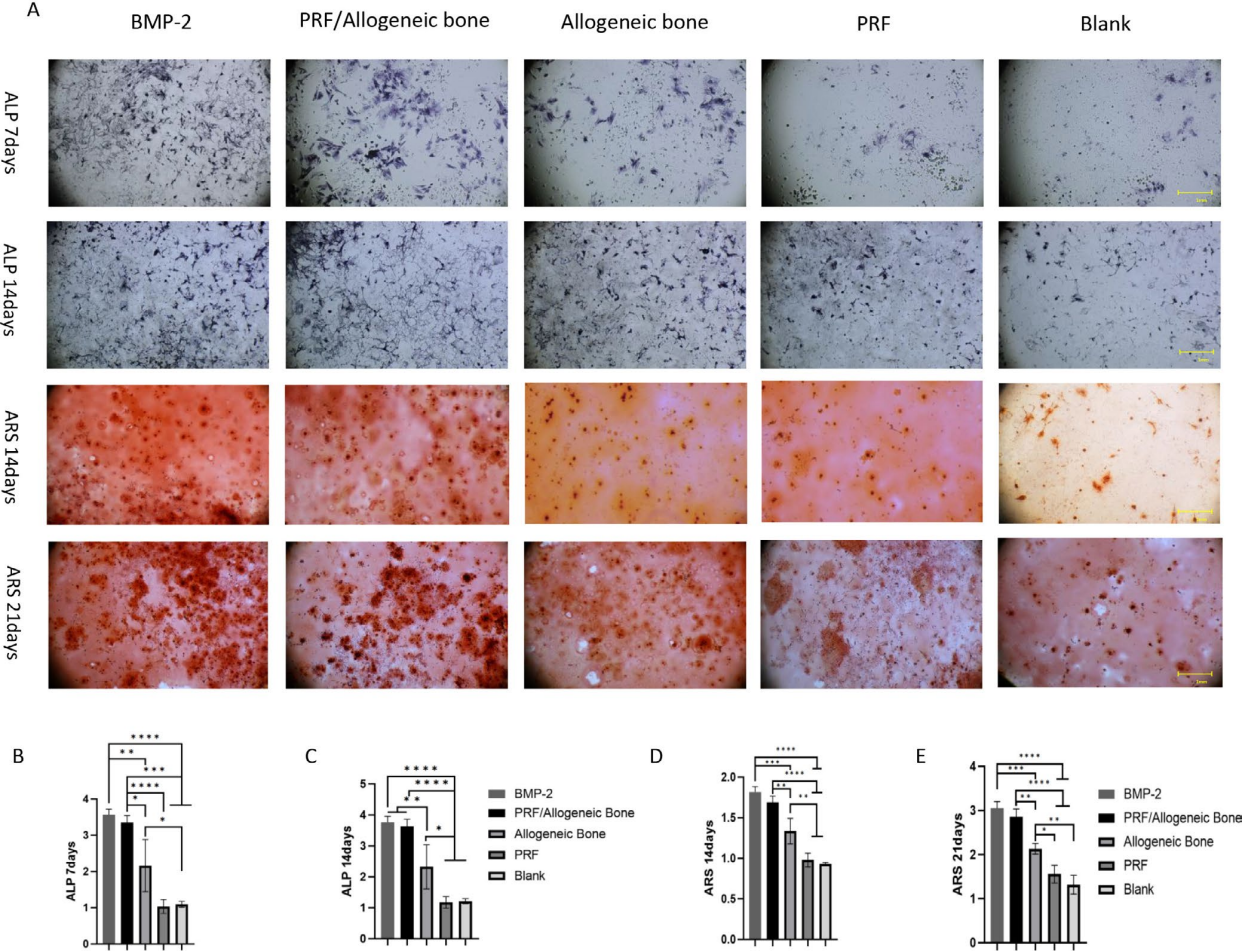
ALP staining and alizarin red S staining. (A) ALP staining and alizarin red staining of different groups. (B, C) The quantitative analyses of ALP expression. (D, E) Quantitative analysis of the ARS results. * indicates significant difference between groups. * indicates significant difference between groups, *p < 0.05, **p < 0.01, ***p < 0.001, and ****p < 0.0001.

Beyond cell proliferation, the osteogenic differentiation of BMSCs is a crucial event for bone regeneration. ALP expression (ALP staining and activity) is recognized as a biomarker of early osteogenic differentiation, involved in the initial processes of new bone formation [10]. BMSCs were still in the early osteogenic stage after 7 days of culture. ALP staining appeared light blue in all extracts, and the ALP‐positive area in the PRF/allograft group extract was larger than in the other groups, indicating a positive effect of PRF on enhancing osteogenic differentiation. Furthermore, quantitative analysis of ALP activity, as shown in Figure 4B,C, revealed that ALP activity in the PRF/allograft group and BMP‐2 group extract was higher than in the remaining groups (*p* < 0.05). However, ALP activity with PRF alone was still lower than with allograft alone (*p* < 0.05), confirming the importance of the combined application of PRF and allograft during the early stage of osteogenesis.

ARS is an indicator of the amount of calcified deposits in BMSCs, representing the later stage of osteogenic differentiation [11]. It was used to assess the osteogenic effect of PRF/bone graft on BMSCs. Calcified nodule deposition in the other groups was greater than in the blank control group (*p* < 0.05). Furthermore, the number of calcified nodules in the PRF/allograft group was similar to that in the BMP‐2 group and significantly higher than in the allograft group and the PRF group (*p* < 0.05) (Figure 4D,E). These results also indicate that the combination of PRF and allograft significantly promoted the formation of mineralized matrix compared to either allograft alone or PRF alone.

### 3.2 In Vivo Osseointegration

No complications such as decreased mobility, partial or complete paralysis were observed in the experimental rabbits that underwent surgery. One rabbit in the autologous bone group died after reduced food intake after surgery. No infection in the surgical area was observed during the entire experimental period. A total of 10 rabbits were included in Group A, 25 in Group B, 40 in Group C, 20 in Group D, and 5 in Group E.

#### 3.2.1 Manual Touch and Imaging Results

**FIGURE 5:**
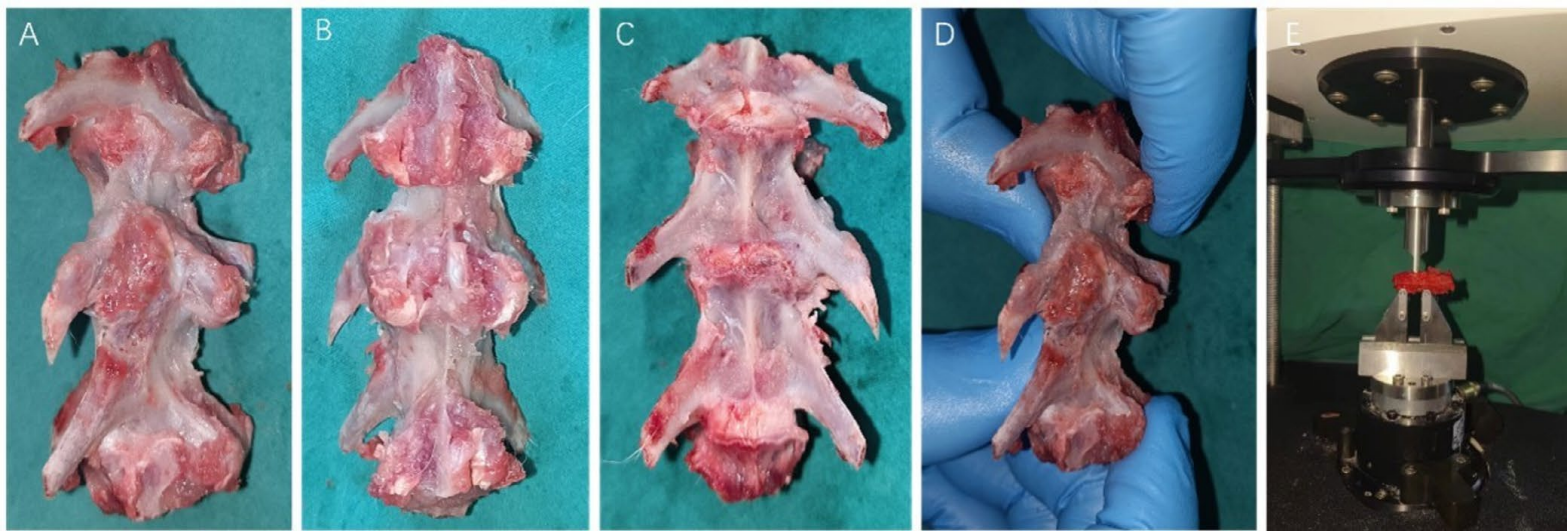
Image of lumbar spine specimen after XLIF surgery. (A) Osteophytes formed on the side of the lumbar spine surgery segment, which wrapped the lateral nail plate (B) Dorsal image of lumbar spine (C) Lumbar spine ventral image showing bony fusion at the surgical level. (D) Checking the effect of surgical segment fusion by manual palpation. (E) Biomechanical machine to detect fusion effect.

**FIGURE 6:**
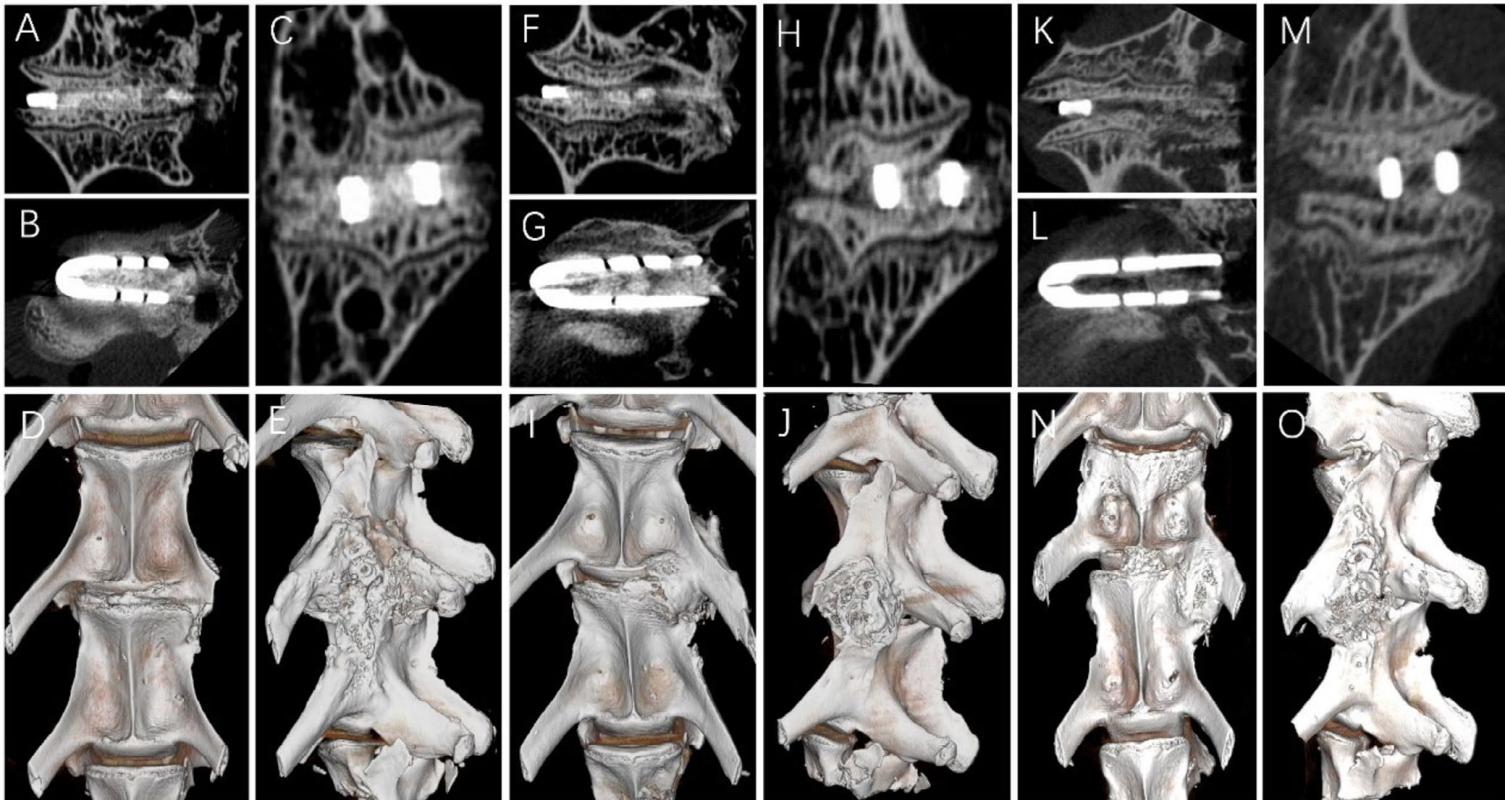
Micro‐CT of the surgical segment was performed 12 weeks after lateral fusion in the rabbit. (A–C) The coronal, axial, and sagittal views of the lumbar spine showed a large amount of new bone formation in the cage. (D) 3D imaging of the ventral lumbar bone showed that the surgical segment had fused. (E) 3D imaging of the lateral lumbar bone showed traces of the screw plate and lateral osteophytes. (F–H) The coronal, axial, and sagittal views of the lumbar spine showed that new bone had grown in the cage, but it had not fused completely, and the lower end plate of the upper vertebra had partially collapsed. (I) 3D imaging of the ventral lumbar bone showed that the surgical segment had not fused completely, and a large amount of osteophytes had been generated on the surgical side. (J) 3D imaging of the lateral lumbar bone showed traces of the screw plate and lateral osteophytes. (K–M) The coronal, axial, and sagittal views of the lumbar spine showed some new bone growth in the cage, but it had not fused. (N) 3D imaging of the ventral lumbar bone showed that the surgical segment had not fused, and a small amount of osteophytes had been generated on the ventral side. (O) 3D imaging of the lateral lumbar bone showed traces of the screw plate and lateral osteophytes.

Figure 5A,B showed that a large amount of osteophytes were generated on the side of the surgical segment of some samples, and the lateral nail plate was wrapped. Figure 5C showed that the intervertebral disk of the adjacent segment on the ventral side of the lumbar spine exists, and the intervertebral space of the surgical segment was bony fused. At 12 weeks after surgery, there was no mobility in Group A and fusion was completed. The fusion rate for specimens in Group B was 70%, with limited mobility, suspected intervertebral fusion but difficult to distinguish. The fusion rate for Group C was 90%. The fusion rate for Group D was 40%. The fusion rate for Group E was 20%, with a certain degree of mobility. Statistically significant differences were observed between Group A and Group D, between Group A and Group E, and between Group C and Group E (*p* < 0.05) (Table 1). All specimens were examined by Micro‐CT, and the quality of intervertebral fusion observed by gross examination was further confirmed by micro‐CT analysis. Figure 6 showed that in rabbits that underwent XLIF, most specimens in groups A, B, C, and D had new bone formation inside and outside the intervertebral cage 12 weeks after surgery. One specimen did not achieve complete fusion 12 weeks after allogeneic bone implantation and PRF. PRF/allogeneic bone was similar to autologous bone, showing obvious ossification and fusion of L4‐L5 vertebrae (Figure 6B,C). No obvious new bone synthesis was observed after fusion cage implantation alone.

**TABLE 1:** Results of manual palpation results 12 weeks postoperation.

	Autologous bone	Allogeneic bone	PRF/allogeneic bone	PRF	Blank	*p*
Fusion/No fusion	10/0 (100%)^#,##^	7/3 (70%)	9/1 (90%)^###^	2/3 (40%)^#^	1/4 (20%)^##,###^	< 0.001*

*Note:* ^#^ *p* = 0.022 versus other indicated treatment, ^##^ *p* = 0.004 versus other indicated treatment, ^###^ *p* = 0.017 versus other indicated treatment, * Indicates statistical significance.

#### 3.2.2 Microcomputed Tomography Analysis and Biomechanical Test

**FIGURE 7:**
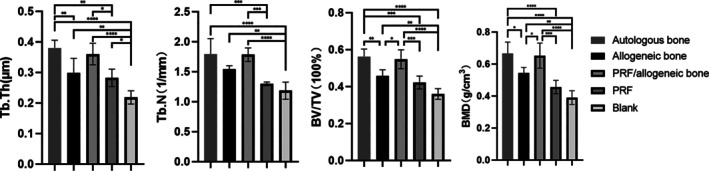
Micro‐CT bone mass measurement. Tb. Th, trabecular thickness; Tb. N, trabecular number; BV, bone volume; TV, tissue volume; BMD, bone mineral density. * indicates significant difference between groups, *p < 0.05, **p < 0.01, ***p < 0.001, and ****p < 0.0001.

The comparison of fusion quality among the fused specimens through quantitative Micro‐CT analysis is shown in Figure 7. The BMD of the newly formed bone in Groups A and C was similar and significantly higher than that in the allogeneic bone group. The new BMD for Group A and Group C was 0.66 ± 0.07 g/cm [3] and 0.65 ± 0.08 g/cm [3], respectively, with no statistically significant difference between them, and both were higher than the other groups (*p* < 0.05). There was no statistically significant difference in BMD between Group D and Group E. The BV/TV for Groups A and C (Group A: 0.56 ± 0.04; Group C: 0.55 ± 0.05) was significantly higher than that of Group B (0.46 ± 0.03) (*p* < 0.001). There was no significant difference in Tb.Th and Tb.N between Groups A and C, but these values were significantly higher than those in Groups D and E (*p* < 0.05).

**FIGURE 8:**
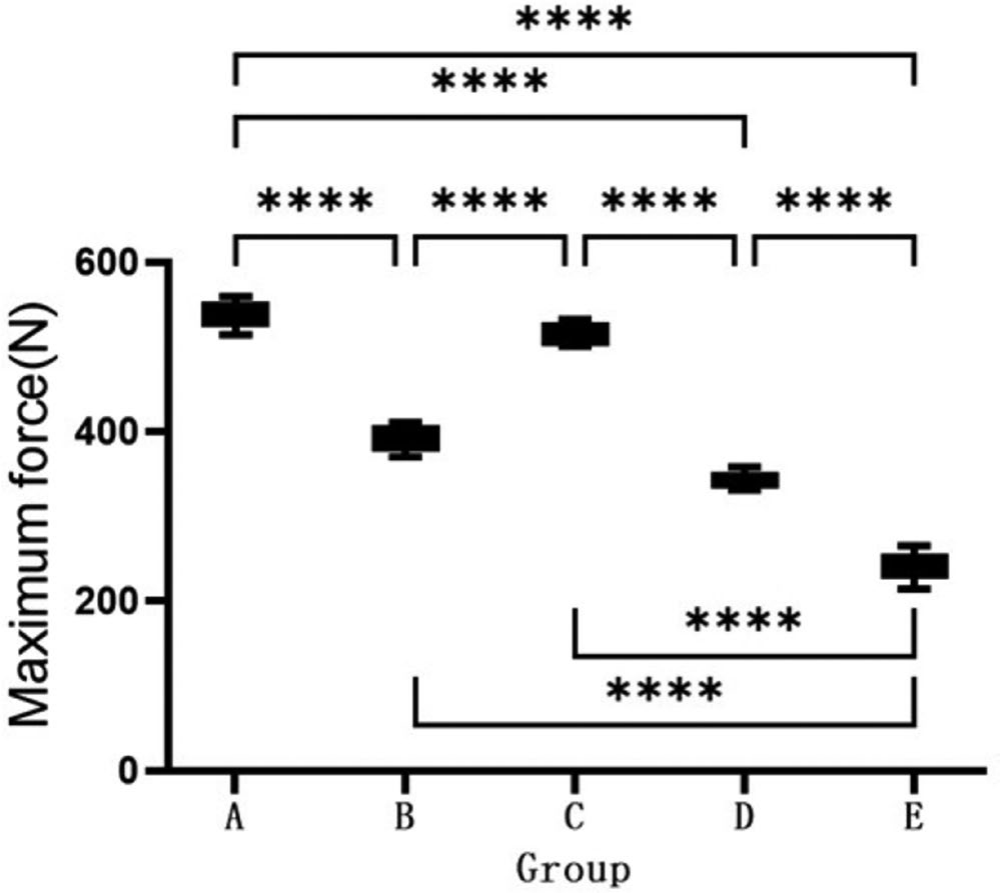
Measurement of maximum force of surgical segment fracture in each group 12 weeks postoperation. * indicates significant difference between groups, *p < 0.05, **p < 0.01, ***p < 0.001, and ****p < 0.0001.

A comparison of the biomechanical parameters is shown in Figure 8. Compared to Groups D and E, the force required to disrupt the fused segment was significantly greater in Groups A and C (A: 540.0 ± 7.87 N; B: 392.2 ± 7.34 N; C: 516.7 ± 6.48 N; D: 342.4 ± 4.89 N; E: 241.8 ± 8.33 N; *p* < 0.05). There was no statistically significant difference in the fracture force of the newly formed bone between Group D and Group E. The maximum fracture force in Groups A and C was significantly higher than that in Group B.

#### 3.2.3 Histological Examination

**FIGURE 9:**
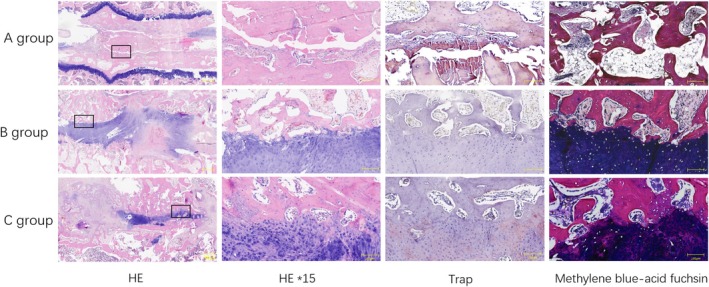
HE, TRAP and methylene blue‐acid fuchsin staining of autologous bone group, allogeneic bone group and PRF/allogeneic bone group at 12 weeks postoperation.

The histological examination of the rabbit spine XLIF specimens was shown in Figure 9. Bone formation occurred in all groups, but in the allogeneic bone group, fibrocartilage tissue existed on the head and tail sides of the new bone, the vertebrae were not completely fused, and the boundaries between them were clear. In the PRF and autologous bone groups, the area of newly formed bone inside and outside the fusion device increased significantly, and fusion was successful. The successful bone integration of the newly formed bone and the vertebrae was further confirmed on the tissue sections. Except for a few discrete areas, the rest had clusters of split chondrocytes and darker stained cartilage tissue. The implanted PRF was completely absorbed in the implant, and there was no obvious histological difference between the PRF and autologous bone groups.

**FIGURE 10:**
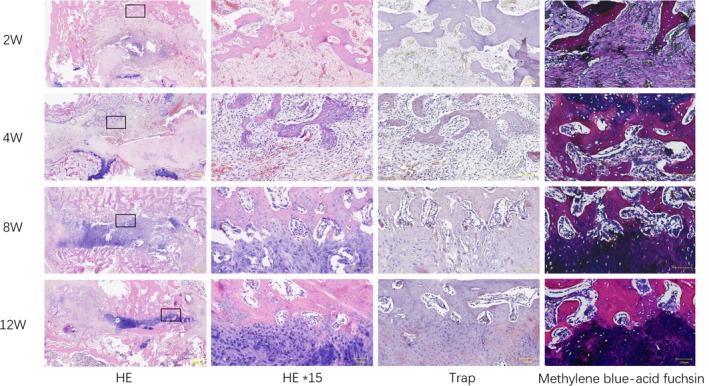
HE, TRAP and methylene blue‐acid fuchsin staining of PRF/allogeneic bone group at 2 W, 4 W, 8 W and 12 W postoperation.

Figure 10 showed that a large number of inflammatory cells and angiogenesis were generated in the PRF/allogeneic bone group 2 weeks after surgery. 4 weeks after surgery, a large amount of fibrocartilage appeared in the intervertebral bone graft area, and the original implanted allogeneic bone was gradually absorbed and reconstructed. 8 weeks after surgery, osteoblasts proliferated in large numbers, and the upper and lower end plates of the intervertebral space in the fusion area grew toward the intervertebral space. Osteoblasts and osteoclasts cooperated with each other to destroy the fibrocartilage and reconstruct trabeculae. Briefly, 12 weeks after surgery, intervertebral bone diffusely proliferated, and the upper and lower end plates were connected by the new bone in the intervertebral space.

#### 3.2.4 ELISA Results

**FIGURE 11:**
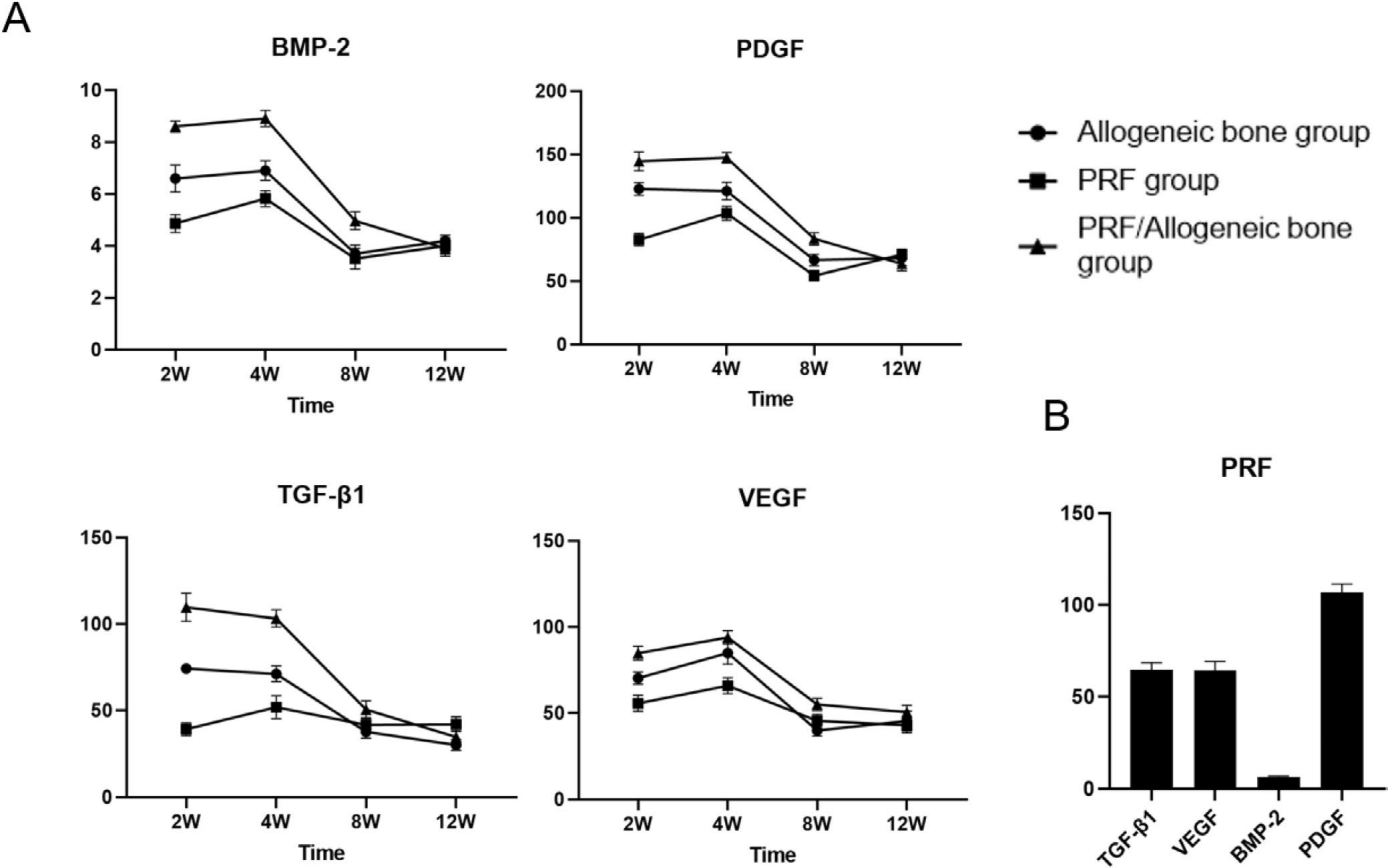
Elisa results of allogeneic bone group, PRF group and PRF/allogeneic bone group at 2, 4, 8 and 12 weeks after surgery A, content of BMP‐2, PDGF, TGF‐β1, VEGF in each group B, content of BMP‐2, PDGF, TGF‐β1, VEGF in prepared PRF.

The concentrations of key osteogenic and angiogenic factors exhibited dynamic changes over time across the groups (Figure 11). At 2 and 4 weeks postoperation, the PRF/Allogeneic Bone group demonstrated significantly higher levels of BMP‐2, PDGF, TGF‐β1, and VEGF compared to both the Allogeneic Bone group and the PRF group (*p* < 0.05). Notably, the levels of these factors measured in the PRF group were lower than those quantified in the prepared PRF material itself, suggesting a potential rapid release and subsequent decrease of cytokines from the PRF after implantation. By 8 weeks, significant differences in BMP‐2 and TGF‐β1 levels persisted specifically between the PRF/Allogeneic Bone group and the Allogeneic Bone group (*p* < 0.05). At the 12‐week endpoint, no statistically significant differences in the levels of these factors were observed among the groups (*p* < 0.05).

## 4 Discussion

In XLIF surgery, achieving rapid and solid fusion is not merely desirable but essential. Autologous bone remains the gold standard grafting material for spinal fusion due to its inherent osteogenic, osteoinductive, and osteoconductive properties. However, XLIF surgery presents a unique challenge: the lateral approach to the intervertebral space fundamentally precludes harvesting sufficient local autologous bone, unlike posterior approaches. Harvesting autologous iliac crest bone requires an additional surgical site, increasing bleeding, operative time, infection risk, and patient morbidity. While allogeneic bone offers logistical simplicity, it carries inherent risks of immune rejection despite processing and critically lacks vital osteogenic cells and osteoinductive proteins. This results in significantly lower osteoconductive activity and fusion efficacy compared to autologous bone, often necessitating extended fusion times and increasing the risk of pseudoarthrosis [12, 13, 14]. Crucially, suboptimal fusion in XLIF can lead to catastrophic complications like endplate subsidence and subsequent loss of disk height, directly undermining the indirect decompression achieved during the procedure and potentially necessitating revision surgery. Therefore, promoting rapid, high‐quality fusion is critical to maintaining disk height stability, preventing implant subsidence, and ensuring the long‐term success of indirect neural decompression. The demonstrated efficacy of the PRF‐allograft composite in achieving fusion strength, density, and rates comparable to autograft directly addresses this core limitation specific to the XLIF technique and mitigates these critical risks.

Autologous platelet concentrates, such as PRP and PRF, are widely used in spinal surgery owing to their high concentrations of growth factors (e.g., PDGF, IGF, EGF, VEGF) that promote bone regeneration [15, 16, 17, 18]. Although PRP is approved for spinal fusion, its efficacy remains debated, potentially due to variable platelet concentrations (where high levels may inhibit bone formation [19]) and the rapid, unsustainable cytokine release triggered by exogenous activators [20]. In contrast, PRF preparation involves no additives, minimizing risks of immune rejection and cross‐infection. Its simple centrifugation yields a stable product rich in growth factors (VEGF, TGF‐β, IGFs, EGF, PDGF) and contains nearly all plasma fibrinogen polymerized into a dense fibrin matrix. This fibrin network acts as a natural scaffold for cell attachment and migration, significantly enhancing bone healing potential and accelerating the fusion process.

In our study, cell viability and proliferation were assessed using Calcein‐AM/PI staining and the CCK‐8 assay, respectively, while cytoskeletal morphology was evaluated via phalloidin/DAPI staining. These assays demonstrated that the platelet‐rich fibrin (PRF), bone graft materials, and titanium alloy cage utilized exhibited no toxicity, possessed excellent biocompatibility, and were suitable for in vivo applications. Furthermore, PRF not only enhanced the biocompatibility of BMSCs but also significantly promoted their proliferative capacity. Results from ALP staining, activity assays, and ARS staining further indicated that the combination of PRF with allograft bone significantly promoted osteogenesis during both early and late stages of osteogenic differentiation. Mechanistically, PRF can activate TGF‐β receptor 1 kinase, induce BMP‐2 production in mesenchymal cells, and directly promote bone regeneration [21]. Studies suggest PRF enhances early osteoblast proliferation and migration via RUNX2, ALP, collagen, and BMP‐2 pathways, thereby significantly increasing the initial vitality and osteogenic potential of the intervertebral fusion site when combined with allograft [22]. Combining PRF with allograft bone provides essential growth factors and a supportive fibrin scaffold, effectively compensating for the deficiencies of allograft alone and creating an environment highly conducive to rapid, robust bone formation.

Previous clinical studies support PRF's potential for promoting fusion. A 2018 study combining PRF matrix, β‐tricalcium phosphate, and bone marrow aspirate achieved posterolateral fusion comparable to autograft [23]. Another study reported a 95% fusion rate at 1 year with PRF/allograft versus 65% with allograft alone [24]. While these imaging‐based studies demonstrate positive effects, they lack early‐stage histopathological analysis. Furthermore, existing small animal models typically simulate posterolateral interbody fusion, failing to accurately model the unique lateral surgical corridor, biomechanics, and specific fusion bed challenges (like endplate loading) inherent to XLIF. To specifically address the clinical context and critical need for reliable fusion in XLIF, we developed a lateral interbody fusion model in New Zealand rabbits meticulously simulating the surgical approach, implant placement, and fusion process of human XLIF [7].

In vivo experiments within a clinically relevant XLIF model demonstrated that PRF mixed with allogeneic bone particles effectively induced robust intervertebral fusion in rabbits. The fusion achieved using this PRF‐allograft composite was biomechanically stronger than fusion achieved with allograft bone particles alone. Notably, PRF promotes the absorption of allograft bone and accelerates the formation of new vertebral bone, ultimately enhancing both the fusion rate and structural integrity. Additionally, the mean bone density and trabecular number within the newly formed bone were significantly higher in the PRF/allograft group compared to the allograft‐only group. Histological analysis revealed in the PRF/allograft group: an inflammatory/angiogenic phase at 2 weeks; robust fibrocartilage formation and active allograft resorption initiation at 4 weeks; massive osteoblast proliferation, endplate growth toward the disk space, fibrocartilage destruction, and trabecular reconstruction at 8 weeks; and near‐complete intervertebral space filling with dense, interconnected trabecular bone bridging the endplates, achieving biomechanical stability by 12 weeks. The ELISA results demonstrated an early peak (2–4 weeks) of key osteogenic and angiogenic factors (BMP‐2, VEGF) in the PRF/Allograft group. This likely reflects the combined effect of osteogenic proteins release from the PRF matrix itself and the subsequent cellular response induced within the fusion bed. While we did not perform in vitro release kinetics, the in vivo temporal profile suggests PRF acts as a sustained reservoir, initiating a robust early biological cascade that compensates for the deficient osteogenic proteins content in processed allograft. This early signal may be critical for accelerating the fusion timeline observed histologically and radiographically.

In addition to its biological advantages, PRF also offers practical benefits for preparation in human spinal surgery. In this study, approximately 1 mL of PRF gel was obtained from 4 mL of rabbit whole blood through a single centrifugation step. The entire preparation process takes less than 15 min and requires minimal manipulation, making it feasible for intraoperative use. In contrast, PRP preparation typically involves two centrifugation steps, the addition of anticoagulants, and activation with calcium chloride or thrombin, which increases procedural complexity, extends preparation time (usually 30–40 min), and elevates the risk of contamination or variability in growth factor release [20, 25]. Moreover, achieving a comparable final product volume generally requires a larger blood volume for PRP [26]. These practical distinctions further support the potential of PRF as a cost‐effective, easy‐to‐handle, and biologically reliable graft enhancer for routine application in XLIF and other spinal fusion procedures.

The application of PRF in spinal fusion, especially XLIF, offers compelling advantages, notably cost‐effectiveness and its critical role in promoting fusion success. Reported 12‐month fusion rates for stand‐alone XLIF range from 85% to 93% [27, 28], with material‐dependent variations: autograft (75%), β‐tricalcium phosphate (89%), and combinations like tricalcium phosphate/iliac crest or BMP‐2 achieving 80%, 89%, and 96.7% at 2 years, respectively [29, 30]. While bone morphogenetic proteins are potent alternatives, they are prohibitively expensive [31]. PRF, derived simply and rapidly (within minutes) from the patient's own peripheral blood under sterile conditions, presents a vastly more economical option. Its autologous origin virtually eliminates immune rejection risk, and the streamlined preparation minimizes infection risk compared to PRP. Considering the evolving landscape of minimally invasive spine surgery and the paramount importance of preventing complications like subsidence in XLIF, achieving high fusion rates is nonnegotiable. These collective findings strongly position the PRF‐allograft composite as a highly promising, cost‐effective alternative to autograft for XLIF procedures. It is capable of achieving comparable, high‐quality fusion efficacy—essential for maintaining disk height, preventing endplate collapse, safeguarding indirect decompression, and ensuring long‐term stability—while simultaneously eliminating the morbidity associated with iliac crest harvest. This solution is uniquely tailored to overcome the grafting limitations and critical biomechanical demands of the lateral approach.

## 5 Limitations

First, due to the small number of samples, it was not possible to quantitatively compare the number of cells with the autologous bone group by immunohistochemistry. Second, we used New Zealand rabbits as a model, and the time required for rabbit bone healing might have been shorter than that for humans. Therefore, it was unclear whether the same fusion effect could be achieved in humans. We would further explore the molecular mechanisms and information pathways of PRF as a bone graft material combined with allogeneic bone to promote intervertebral fusion.

## 6 Conclusions

The combination of PRF and allogeneic bone significantly promoted intervertebral fusion in a rabbit model of XLIF, and the fusion strength was similar to that achieved using autogenous bone. The results of this study indicated that PRF might become a new bone grafting method for treating patients with spinal diseases in the future.

## Author Contributions

W.W. and H.W. performed the most experiments. J.L. conducted part of the in vitro experiments. Statistical analysis was performed by S.L. The manuscript was written by W.W. W.Z. and S.Y. contributed to the study conception and supervised the project. W.Z. revised and polished the manuscript. All authors have read and approved the final manuscript.

## Funding

This study was supported by S&T Program of Hebei 22377708D; Hebei Provincial Government funded Provincial Medical Talent Project; Hebei Medical University “14th Five‐Year Plan” Clinical Medical Innovation Research Team.

## Conflicts of Interest

The authors declare no conflicts of interest.

## Supporting information


**Table S1:** Diagram of Study Groups.

## Data Availability

The data that support the findings of this study are available from the corresponding author upon reasonable request.
