# Supplementary material for: Platelet‐Rich Fibrin Induces New Bone Formation to Promote Extreme Lateral Interbody Fusion of Spine in Rabbits
Source: JOR Spine. 2026 Apr 15;9(2):e70179. doi: 10.1002/jsp2.70179 (PMC13081695; doi:10.1002/jsp2.70179)
Supplement: Supplementary file 1 — Table S1: Diagram of Study Groups. [file JSP2-9-e70179-s001.docx]

Table S1. Diagram of Study Groups

| Group (n total) | Day of Observation | n | Test |
| --- | --- | --- | --- |
| A (10 rabbits) | 12W (Manual Palpation)  n=10 | 5 | CT, Histology |
|  |  | 5 | Biomechanics |
| B (25 rabbits) | 2、4、8W（each point n=5） | 15 | ELISA |
|  | 12W（Manual Palpation）  n=10 | 5 | CT, Histology |
|  |  | 5 | Biomechanics, ELISA |
| C (40 rabbits) | 2、4、8W（each point n=10） | 15 | ELISA |
|  |  | 15 | Histology |
|  | 12W（Manual Palpation）  n=10 | 5 | CT, Histology |
|  |  | 5 | Biomechanics, ELISA |
| D (20 rabbits) | 2、4、8W（each point n=5） | 15 | ELISA |
|  | 12W（Manual Palpation）  n=5 | 5 | CT, Biomechanics, ELISA |
| E (5 rabbits) | 12W（Manual Palpation）  n=5 | 5 | CT, Biomechanics |
